# Psoriatic arthritis successfully treated with second-line anti-interleukin-6 treatment: a case report and review of the literature 

**DOI:** 10.1186/s13256-022-03624-z

**Published:** 2022-11-03

**Authors:** Tatsuhiko Kutsuna, Kazunori Hino, Hitoshi Hasegawa, Kunihiko Watamori, Teruki Kidani, Hiroshi Imai, Hiromasa Miura

**Affiliations:** 1 Department of Bone and Joint Surgery, Ehime University Graduate School of Medicine, Shitsukawa, Toon, Ehime 791-0295 Japan; 2 Department of Joint Reconstruction, Ehime Graduate School of Medicine, Toon, Ehime Japan; 3 Department of Hematology, Clinical Immunology and Infection Diseases, Ehime University Graduate School of Medicine, Toon, Ehime Japan

**Keywords:** Psoriatic arthritis, Clinical remission, TNF failure, Anti-IL-6, Artificial joint replacement, Biologics

## Abstract

**Background:**

Psoriatic arthritis treatment with antitumor necrosis factor has been shown to reduce disease activity. Nonetheless, more than 30% of patients do not achieve a sufficient response to tumor necrosis factor blockers. Currently, treatment with interleukin-6 inhibitors is expected to be effective and suppress the joint destruction in patients with psoriatic arthritis; however, evidence regarding their efficacy is limited to a few reports.

**Case presentation:**

A 78-year-old Japanese woman with psoriatic arthritis associated with rapid joint destruction was successfully treated with a second-line anti-interleukin-6 receptor agent. In this case, a tumor necrosis factor inhibitor induced an inadequate response, and the right knee and left hip joints required artificial joint replacement surgery. However, second line treatment with anti-interleukin-6 treatment was effective, and the right elbow joint function was preserved.

**Conclusions:**

We experienced a case of psoriatic arthritis, in which anti-interleukin-6 treatment repaired a bone cyst in the lateral epicondyle of the humerus and enthesitis of the distal interphalangeal joints. The patient is currently in clinical remission with no restrictions in daily life activities. Anti-interleukin-6 treatment may address the unmet needs of patients with psoriatic arthritis who are resistant or intolerant to antitumor necrosis factor treatment, with rapidly destructive large joints but with well-managed skin manifestations.

Psoriatic arthritis (PsA) is an inflammatory arthritis condition associated with psoriasis [1]. A previous report emphasized a benign course in most patients [2]; however, PsA often leads to impaired function and reduced quality of life [3, 4]. The 2016 European League Against Rheumatism recommends that treatment escalation is relevant if there is evidence of active arthritis regarding swollen joints and/or at least moderate disease activity, as evaluated by the joint condition (the number of swollen and tender joints) and an inflammatory reaction based on laboratory examinations of C-reactive protein (CRP) and erythrocyte sedimentation rate [5]. Tumor necrosis factor (TNF) inhibitors are well established to be safe and efficacious for the treatment of PsA with skin and joint involvement, and to prevent radiographic damage [5].

In the present case, TNF inhibitor [adalimumab (ADA)] therapy was ineffective, and the right knee and left hip joints required artificial joint replacement. However, the second-line anti-interleukin-6 (IL-6) [tocilizumab (TCZ)] treatment was effective, and the right elbow joint function was preserved.

## Case presentation

**Fig. 1:**
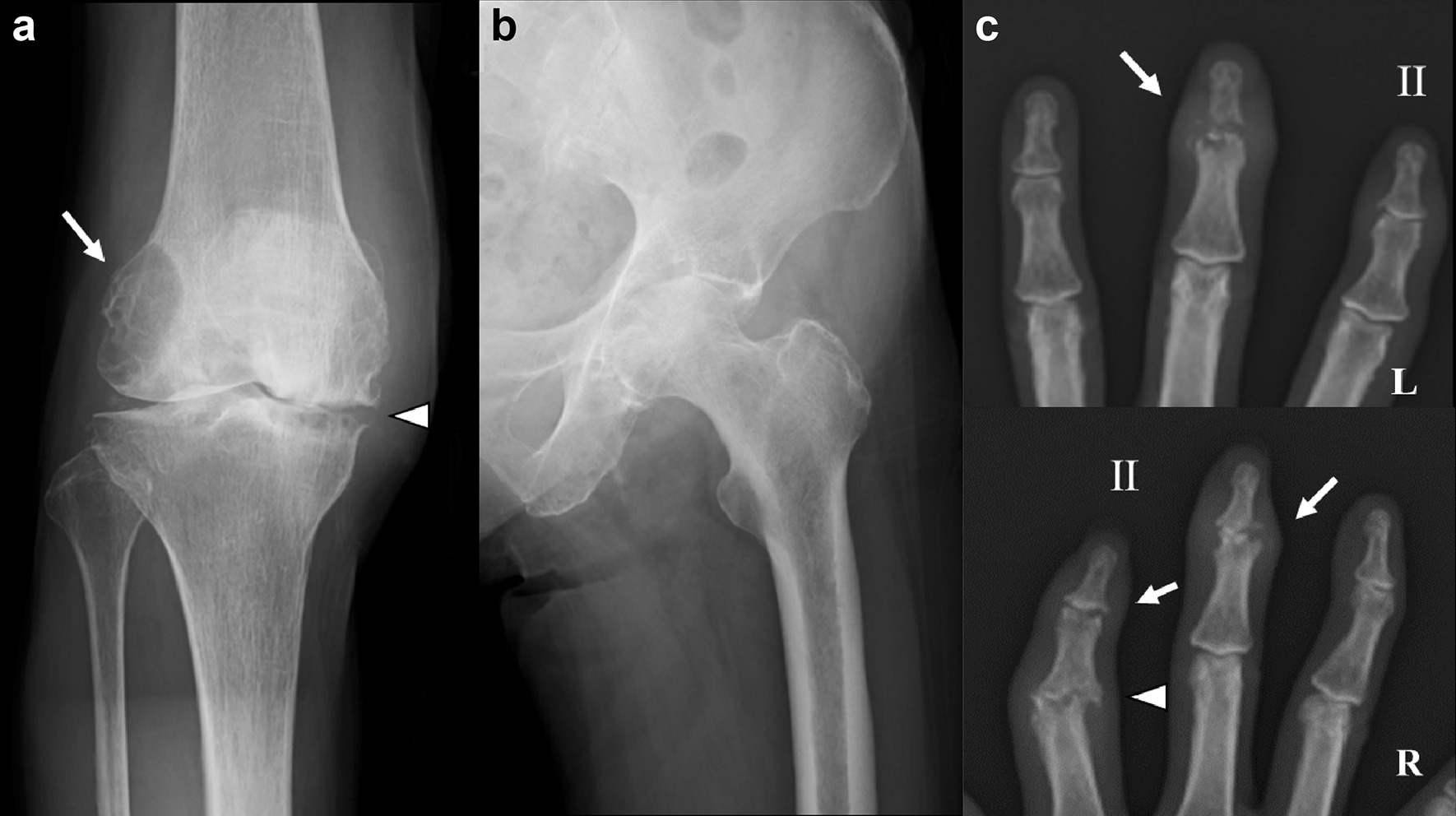
X-ray findings at the first visit. **a** Anteroposterior radiograph of the knee. Bone cyst formation in the lateral condyle of the femur (arrow) and joint destruction of the medial compartment (arrowhead). **b** Narrowing joint space of the left hip. **c** Pencil cup-like destruction of the distal interphalangeal joint (arrow) and destruction of the proximal interphalangeal joint (arrowhead)

**Fig. 2:**
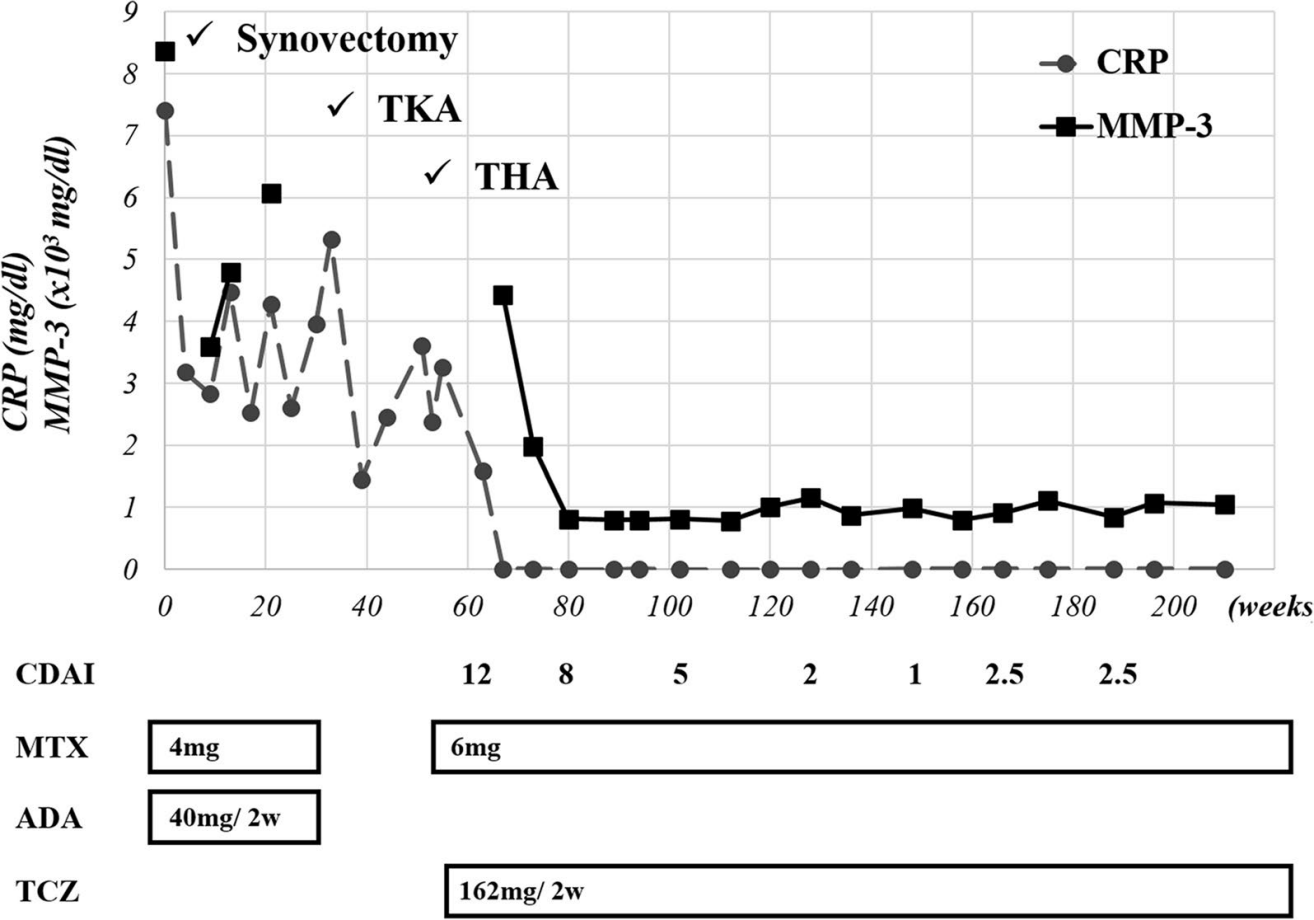
The clinical course of this case. *TKA* total knee arthroplasty, *THA* total hip arthroplasty, *CDAI* clinical disease activity index, *MTX* methotrexate, *ADA* adalimumab, *TCZ* tocilizumab

**Fig. 3:**
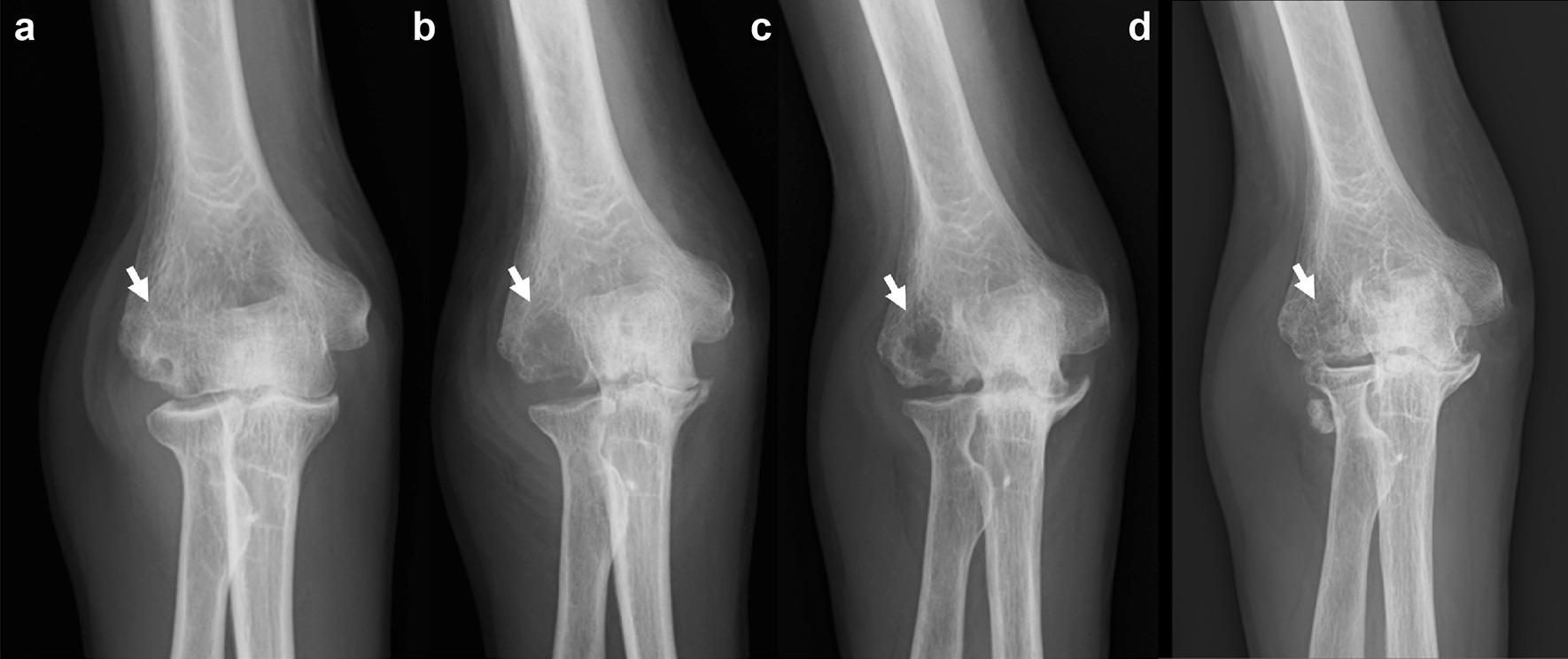
The time course of bone cyst in the right elbow (arrows). **a** First visit at our hospital. **b** Before tocilizumab. **c** One year after tocilizumab. **d** Six years after tocilizumab (arrows indicate the bone cyst)

**Fig. 4:**
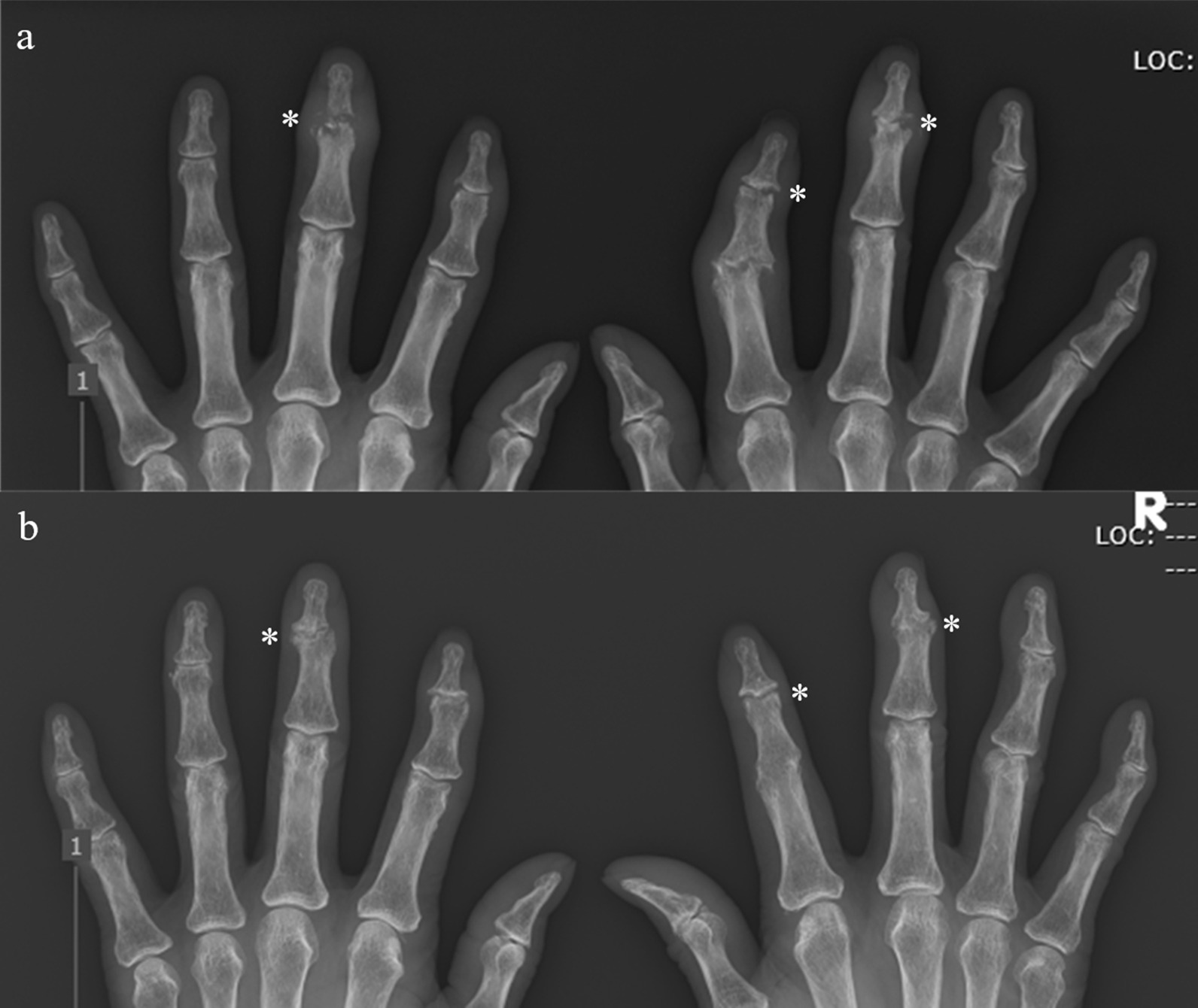
The time course of the anteroposterior radiograph of the distal interphalangeal joint (asterisk). **a** First visit at our hospital. **b** Six years of tocilizumab treatment

A 78-year-old Japanese woman presented to our hospital with severe bilateral knee pain (right > left) for 14 months. At the first visit to the outpatient department, evidence suggestive of polyarthritis and a rash on the right knee were observed. Polyarthritis was indicated by three swollen joints (bilateral knee and right elbow) and six tender joints [bilateral distal interphalangeal (DIP) joints of the middle finger, right proximal interphalangeal (PIP) joint of the index finger, bilateral knee, and right elbow]. Before presenting to our hospital, the patient had a history of psoriasis vulgaris for which she received outpatient treatment for 1 year; however, she had been in clinical remission and had completed the follow-up. Psoriasis vulgaris recurrence was ruled out by a skin biopsy performed on the rash of the right knee. On the day of her presentation as an outpatient, laboratory data showed negative results for both rheumatoid factor and anti-citrullinated peptide antibody; however, CRP (4.47 mg/dL) and matrix metalloprotease (MMP)-3 (836.5 ng/mL) levels were abnormally high. Radiographic examination showed bone cyst formation in the lateral condyles of the femur, disappearance of the bilateral knee joint space, narrowing of the left hip joint space, pencil-in-cup-like joint destruction, juxtaarticular new bone formation of the DIP, and joint destruction of the PIP (Fig. 1). Magnetic resonance imaging was performed at 1 week after the first consultation at our hospital. Low-intensity T1-weighted and high-intensity T2-weighted images showed an intraosseous cystic lesion in the lateral condyle of the left femur, and a contrast effect was observed at the margin of the cyst. The pathological findings at synovectomy of the right knee, performed 6 months after the outpatient presentation, were only suggestive of a chronic inflammation pattern. We diagnosed PsA based on Classification Criteria for Psoriatic Arthritis (CASPAR) score of 4 points (skin psoriasis, previously present; negative rheumatoid factor; history of dactylitis; and juxtaarticular new bone formation on the radiograph) [6]. Although treatment with methotrexate (MTX) at 4 mg/week and ADA at 40 mg/2 weeks was initiated, the patient’s joint destruction progressed and activity of daily living was severely restricted, indicating poor control of disease activity. Following severe restriction of activities of daily living owing to the right knee and left hip pain, sequential total knee and total hip arthroplasty were performed at an interval of 4 months, and MTX at 6 mg/week was recommenced at 4 weeks postoperatively. The right elbow joint pain persisted, and radiography showed bone cysts at the lateral humeral condyle in the right elbow. After 8 weeks of using MTX alone, we decided to add TCZ at 162 mg/2 weeks as a second-line biologic. At that time, inhibitors of interleukin (IL)-22/23 and 17 had not yet obtained insurance approval in Japan. At 4 weeks after initiation of TCZ treatment, the right elbow joint pain and swelling were improved. At 8 weeks after TCZ initiation, MMP-3 gradually decreased (198.7 ng/mL). Twelve weeks after TCZ initiation, MMP-3 improved further (81.1 ng/mL), and low disease activity [clinical disease activity index (CDAI) < 10] was observed. Thereafter, CDAI decreased further and the patient achieved the clinical remission rate (CDAI <2.8) at 1 year after TCZ initiation (Fig. 2). In addition, radiographs showed repair of the bone cyst at the lateral humeral condyle in the right elbow and repair of the DIP joints (Figs. 3, 4). The patient has been in clinical remission for 5 years, with no restrictions of daily life activities.

## Discussion and conclusion

PsA treatment with anti-TNF agents has been shown to reduce disease activity parameters and radiographic progression, and is effective in the treatment of skin lesions and enthesitis, while improving quality of life [7–9]. Nonetheless, more than 30% of PsA patients do not respond sufficiently to TNF blockers [10].

Newer biologic drugs target IL-12/23 and IL-17, the upstream inflammatory cytokines responsible for macrophage activation that lead to increased TNF and IL-6 secretion [11]. These drugs have shown their efficacy in clinical trials for PsA [12, 13]. At the time of treatment in the present case, the new biologic drugs were not yet available in Japan; hence, we selected an IL-6 inhibitor as the second-line biological disease-modifying antirheumatic drug (bDMARD).

In the pathology of PsA, T-helper (Th) 17 cells and the associated IL-23/IL-17 axis are crucial. IL-17 drives synovial fibroblasts and macrophages to promote secretion of inflammatory cytokines, such as IL1-β, IL-6, and TNF-α, resulting in bone destruction [14]. Moreover, IL-6 induces differentiation of naive T-cells into Th17 cells, further increasing these inflammatory cytokines [15]. High levels of IL-6 have been found in psoriatic skin lesions and the synovial tissue of patients with PsA [16]. Furthermore, serum IL-6 levels positively correlated with the Disease Activity Score-28 based on CRP level in patients with PsA [17]. These findings make IL-6 a potential therapeutic target in PsA.

Meese *et al*. reported the results of an exploratory phase 2 study with the IL-6 inhibitor clazakizumab in PsA [18]. They revealed that treatment using clazakizumab significantly improved musculoskeletal manifestations (joint signs and symptoms, enthesitis, and dactylitis), with minimal improvements of skin disease compared with placebo. They argued that some PsA patients do not present skin lesions at diagnosis but may benefit from IL-6 treatment [19]. Additionally, there have been a few reports describing successful outcome using anti-IL-6 receptor biologic therapy to treat PsA [19, 20]. TCZ is an IL-6 receptor antibody that differs in mechanism from clazakizumab, which directly inhibits serum IL-6. TCZ inhibits IL-6 production indirectly by interfering with IL-6 binding to its receptor, which causes unbound IL-6 to accumulate in the serum [21]. After the abrogation of immune activation, IL-6 serum levels gradually decrease [21]. We considered that this was the reason that the treatment required 6 months to induce clinical remission in the present case. Winchester *et al*. suggested non-overlapping mechanisms of inflammation in the skin and joints of patients with PsA, as evidenced by differences in genetic profiles in patients with psoriasis compared with those with PsA [22].

Currently, IL-12/23 and IL-17 inhibitors should be prioritized as second-line bDMARDs [5]. Nonetheless, we experienced a case in which TCZ repaired a bone cyst in the lateral epicondyle of the humerus and enthesitis of the DIP joints. The patient has been in clinical remission with no restrictions on daily life activities. TCZ may address the unmet needs of patients who are PsA resistant or intolerant to the anti-TNF treatment with rapidly destructive large joints in whom skin manifestations are well managed.

## Data Availability

The datasets used in the current study are available from the corresponding author on reasonable request.

## Abbreviations

IL: Interleukin
PsA: Psoriatic arthritis
CRP: C-reactive protein
TNF: Tumor necrosis factor
ADA: Adalimumab
TCZ: Tocilizumab
DIP: Distal interphalangeal
PIP: Proximal interphalangeal
MMP-3: Matrix metalloprotease-3
CASPAR: Classification Criteria for Psoriatic Arthritis
MTX: Methotrexate
CDAI: Clinical disease activity index
bDMARD: Biological disease-modifying antirheumatic drug
Th: T-helper
